# Digital Sexual Violence and Suicide Risk in a National Sample of Sexual Minority Adolescents

**DOI:** 10.1177/08862605221116317

**Published:** 2022-08-09

**Authors:** Ankur Srivastava, Joshua Rusow, Sheree M. Schrager, Rob Stephenson, Jeremy T. Goldbach

**Affiliations:** 1 School of Social Work, University of North Carolina, Chapel Hill, NC, USA; 2 Department of Research on Children, Youth, and Families, Children’s Hospital Los Angeles, Los Angeles, CA, USA; 3 Office of Research and Sponsored Programs, California State University Northridge University Northridge, CA, USA; 4 Department of Systems, Populations and Leadership, and The Center for Sexuality and Health Disparities, School of Nursing, University of Michigan, Ann Arbor, USA; 5 Brown School of Social Work, Washington University in St. Louis, St. Louis, MO, USA

**Keywords:** sexual minority adolescents, digital sexual violence, online violence, nonconsensual, suicide, self-harm

## Abstract

This paper aimed to examine the association between digital sexual violence (threat to post or nonconsensual posting of sexually explicit media) and suicidal (ideation, planning, and attempt) and non-suicidal self-harm behavior. The data for the current analysis come from an online sample of sexual minority adolescents (aged 14–17) recruited from across the United States (*n* = 970). Multivariate logistic regressions were used to examine the association between digital sexual violence with suicide (ideation, planning, and attempt) and self-harm. In the sample, 9.1% of participants reported being threatened to have their sexually explicit media posted without their consent, while 6.5% reported their sexually explicit media had been posted without their consent. Threat to post sexually explicit media without consent was associated with higher odds of reporting suicidal ideation (odds ratio [OR] = 1.88), suicide plan (OR = 2.12), suicide attempt (OR = 3.56), and self-harm (OR = 1.96). While nonconsensual posting of sexually explicit media was associated with higher odds of reporting suicidal ideation (OR = 1.82) and suicide attempt (OR = 2.20). All models controlled for age, assigned sex at birth, sexual identity, and race and ethnicity. These findings underscore important considerations and future research directions. Given the associations between digital sexual violence and suicide risk among sexual minority adolescents, suicide prevention efforts with adolescents must be responsive to the needs of sexual minority adolescents and the changing landscape of sexual violence in digital spaces. Future research should examine the trajectories of digital sexual violence among adolescents and comparative analyses by demographic subgroups to better understand changes in these processes over time.

The last decade has seen increased public and academic attention on sexting—loosely defined as sharing sexually explicit media through text messages and other electronic platforms (Klettke et al., 2014; Krieger, 2017; Madigan et al., 2018; Ringrose et al., 2022). In particular, the phenomenon is seen with an upward trend among adolescents (Del Rey et al., 2019; Mitchell et al., 2012). A systematic review reported the prevalence of sexting among adolescents (aged 10–19 years) was 10.2% for sending and 15.6% for receiving sexually explicit content (Klettke et al., 2014).

A more recent emerging and concerning phenomenon is nonconsensual sharing, whereby sexually explicit media (images or videos) are shared beyond the intended receiver without consent, which has been identified as a new form of digital sexual violence (Bindesbøl Holm Johansen, 2019; Ringrose et al., 2013). This form of violence often includes issues of sextortion (i.e., to coerce a person in unwanted sexual behavior via sexually explicit media) or revenge porn (i.e., sharing or threat of sharing of sexually explicit media without the sender’s consent; Barak, 2005; Henry & Powell, 2018). A systematic review on nonconsensual sharing of sexually explicit media found that among adolescents, rates ranged between 1.5% and 32% (Walker & Sleath, 2017).

One group that consistently reports high rates of digital sexual violence is sexual minority adolescents (Van Ouytsel et al., 2019). For example, in a representative sample of high school students, sexual minority students had three times the odds of engaging in sexting than their heterosexual counterparts (Rice et al., 2012). Similarly, Priebe and Swedin (2012) reported that sexual minority youth in their study had increased odds of having sexually explicit content shared without consent (sixfold odds among participants assigned male sex at birth and twofold odds among participants assigned female sex at birth) compared to their heterosexual peers. In addition, studies have also reported sexual minority adolescents are twice as likely to be the victim of sextortion (10.5% vs. 4.5%; Patchin & Hinduja, 2020) and sexting under pressure (37.5% vs. 19.6%) than their heterosexual peers (Van Ouytsel et al., 2021).

Although several studies have explored the associations between digital sexual violence and suicidality in general populations of youth (Medrano et al., 2018; Wachs et al., 2021), no studies have examined this phenomenon in sexual minority adolescents. This is particularly concerning, because studies (including meta-analyses) continuously find this population at much greater risk of suicide and non-suicidal self-harm (Blashill et al., 2021; Fulginiti et al., 2020; Marshal et al., 2011; Taliaferro et al., 2017). Recent studies have reported that sexual minority adolescents had higher lifetime risk of suicidal ideation (48% vs. 13%), plan (16.6% vs. 5.4%), attempt (12.0% vs. 5.4%), and self-harm (29.7% vs 10.6%) compared to their heterosexual peers (Kann et al., 2018; Liu et al., 2019; Luk et al., 2021; Taliaferro et al., 2017). Relatedly, studies have found a positive association between general cybervictimization and suicide risk in this population (Bishop et al., 2021; Cénat et al., 2015; Duong & Bradshaw, 2014; Sinclair et al., 2012). However, the association between suicide risk and digital sexual violence (nonconsensual sharing of sexually explicit media) among sexual minority adolescents has yet to be explored.

## Current Study

Frameworks such as minority stress theory (Meyer, 2003) posit that these high rates of violence and victimization (i.e., distal stressors) can lead to increased rates of behavioral health outcomes including risk for suicide and self-harm (Fulginiti et al., 2020; Smith et al., 2020). For example, Nydegger and colleagues (2020) found that sexual minority identity was a moderated association between dating violence and suicide risk. Given suicide disparities and increased rates of digital sexual violence among sexual minority adolescents, the present study examined how experiences of nonconsensual sharing of sexually explicit media are related to suicide and self-harm in a national sample of sexual minority adolescents (*N* = 970). We asked the following research question: what is the association between digital sexual violence and suicide risk (both suicidal and non-suicidal self-harm behavior)? Given the existing literature, we hypothesized that there will be a positive association between digital sexual violence and suicide risk in our sample. Understanding the relationship between digital sexual violence and suicide and self-harm among sexual minority adolescents has the potential to inform the content of interventions that aim to improve the mental health of sexual minority adolescents.

## Method

### Participants and Procedures

The data for the current analysis come from a larger study of a national online sample of sexual minority adolescents, which enrolled 1,076 adolescents and followed them over 3 years with seven time points, to examine pathways that may predict differing behavioral health outcomes in sexual minority adolescents. The online sample of sexual minority adolescents was recruited from across the United States via targeted social media advertising (Facebook, Instagram, YouTube) based on race, ethnicity, geography, and urbanicity. A brief screener determined the study eligibility (aged 14–17, identified as cisgender [sex a ssigned at birth matches current gender identity], provided a U.S. ZIP code, and reported a sexual attraction other than heterosexual or straight at baseline). The parent study was restricted to cisgender participants as one of the primary predictors of the study (i.e., minority stress) was measured through a robust set of scales that had yet to be validated with transgender and gender nonbinary adolescents. Subsequent studies have since validated the measure with transgender and gender nonbinary adolescents, but at the time this lack of empirical data led to more restrictive inclusion criteria. To ensure data integrity, numerous checks for fraud (e.g., duplicate email address or contact information, screening out on first attempt and re-entering with false responses to get through the screener) and data quality (e.g., unrealistic survey completion times, low validation scores based on attention check measures, or decline to answer numerous questions) were completed before respondents were included in the final baseline data. Participants considered to be nonfraudulent were invited to the longitudinal study and had the opportunity to refer up to three other adolescents to the study. The referred participants went through the same fraud and data quality checks before entering the study. All participants provided online assent prior to completing the survey. The data for this paper come from time point 2 (6-month follow-up, *n* = 970), when a question regarding nonconsensual threatening and posting of sexually explicit media was introduced. Participants received $20 for their participation in this wave of the study (refer Schrager et al., 2022 for detailed study protocols). All study methods were approved by the University of Southern California Social-Behavioral Institutional Review Board.

### Measures

#### Demographics

Demographic characteristics (age, race and ethnicity, sex assigned at birth, and sexual identity) were measured as follows: race and ethnicity item had six response options (Native American, American Indian, or Alaska Native; Asian or Pacific Islander; Black or African American; White; Latino or Hispanic; and race and ethnicity not listed); respondents could choose all categories with which they identified. Participants who chose multiple racial and ethnic categories were coded as multi-racial. To assess sex, participants were asked “What was your sex assigned at birth?” Response options were “male” and “female.” Sexual orientation and identity was assessed by asking an open-ended question, “What would you say is your sexual orientation or identity?” The research team used prior work with sexual identity variables and responses to this question to design a qualitative coding scheme. The responses were coded as gay, lesbian, bisexual, pansexual, complex or multiple identities (e.g., gay pansexual, bisexual lesbian), queer, straight or mostly straight, asexual, and another identity (e.g., demisexual, agrosexual). For analytic purposes, this variable was collapsed into four categories: (a) gay or lesbian, (b) bisexual, (c) pansexual, and (d) complex or multiple or another identity.

#### Digital sexual violence

Digital sexual violence was measured using items adapted from Ruvalcaba and Eaton (2020). Participants were asked about being threatened (“Someone threatened to send and/or post photos, images, or videos [of me] with intimate or sexual media to others without my permission”); and if such media were posted (“Someone sent and/or posted photos, images, or videos [of me] with intimate or sexual media to others without my permission”). Participants were also asked if they were threatened, or the media was posted by their partners (“Was the person who shared that media a romantic partner [either current or former]?”). In addition, they were asked if they have been the perpetrator and threatened someone or posted explicit media of someone else without their consent. Response options for all the questions were: “yes,” “no,” and “decline to answer.”

#### Suicide risk and self-harm

Suicide risk was assessed with items adapted from the Columbia-Suicide Severity Rating Scale and the Suicide Behaviors Questionnaire-Revised (Osman et al., 2001; Posner et al., 2011). Adapted items from the former assessed presence of ideation (“During the past 6 months, did you ever seriously consider attempting suicide?”); planning (“During the past 6 months, did you make a plan about how you would attempt suicide”); and attempt (“How many times did you actually attempt suicide?”). Response options for ideation and planning were: “yes,” “no,” and “decline to answer.” Suicide attempts were recorded as follows: 0, 1, 2, 3, 4, 5, and 6 or more. For analyses, attempts were recoded as 0 (*no attempt*) and 1 (*made an attempt*). Participants were also assessed regarding self-harm: “During the past 12 months, how many times did you do something to purposely hurt yourself without wanting to die, such as cutting or burning yourself on purpose?” Response options were as follows: 0, 1, 2, 3, 4, 5, and 6 or more. For analyses, self-harm was recoded as 0 (*absence*) and 1 (*presence*). The decision to recode suicide attempt and self-harm as dichotomous were based on the frequency distribution on these variables.

### Data Analysis

All four outcome variables (suicidal ideation, plan, attempt, and self-injury) were coded as dichotomous to represent presence (coded as 1) or absence (coded as 0) of the construct. A series of multivariate logistic regression models were run to examine the association of digital sexual violence with suicide (ideation, planning, and attempt) and self-harm. The results from the analyses are presented by type of digital sexual violence—threat to post sexually explicit media and nonconsensual posting of sexually explicit media. All models controlled for age, assigned sex at birth, sexual identity, and race and ethnicity. Data were analyzed using Stata 14.2 (Long & Freese, 2006).

## Results

### Sociodemographic Characteristics

Sample characteristics are presented in Table 1. The average age of the sample was 16.3 years (SD = 1.1); most participants reported sex assigned at birth as female (68.3%, *n* = 662). Of the sample, 58% (*n* = 562) identified as White or Caucasian, followed by Latino or Hispanic (13.4%, *n* = 130); multi-racial (10.6%, *n* = 103); Asian, Pacific Islander, Native American, American Indian, or Alaska Native (9.9%, *n* = 96); and Black or African American (8.1%, *n* = 79). In terms of sexual identity, 38.5% (*n* = 373) identified as bisexual, followed by gay or lesbian (38.0%, *n* = 369), pansexual (12.8%, *n* = 124), and complex or multiple identities (10.7%, *n* = 104).

**Table 1.** Sample Characteristics (*N* = 970).

	*n* (%) or *M* (SD)
Age (range 14–17)	16.34 (1.09)
Male	308 (31.8%)
Female	662 (68.3%)
Race	
White/Caucasian	562 (57.9%)
Latino/Hispanic	130 (13.4%)
Black or African American	79 (8.1%)
Asian/Pacific Islander	68 (7.0%)
Native American/American Indian	28 (2.9%)
Multi-racial or multi-ethnic	103 (10.6%)
Sexual Identity	
Gay/Lesbian	369 (38.0%)
Bisexual	373 (38.5%)
Pansexual	124 (12.8%)
Complex/Multiple/Another Identity	104 (10.7%)
Digital Sexual Violence	
Nonconsensual sharing (victim)	
Threat	88 (9.1%)
Threat (from partner)	32 (36.8%)
Posted	63 (6.5%)
Posted (from partner)	23 (35.5%)
Nonconsensual sharing (perpetrator)	
Threat	5 (0.5%)
Posted	21 (2.2%)
Suicide Risk	
Ideation	39.7%
Plan	23.2%
Attempt	10.8%
Self-harm	43.4%

*Note.* Digital sexual violence = threatened or posted intimate or sexual content (photos, images, or videos) without one’s consent.

### Digital Sexual Violence

In the sample, 9.1% (*n* = 88) of participants reported being threatened to have their sexually explicit media posted without their consent; of those, 36.8% (*n* = 32) were threatened by their current or former partner. Similarly, 6.5% (*n* = 63) reported their sexually explicit media had been posted without their consent; of those, 35.5% (*n* = 23) reported the media been posted by their current or former partner. In addition, 0.5% (*n* = 5) of participants reported threatening and 2.2% (*n* = 21) reported posting sexually explicit media of someone without their consent.

### Digital Sexual Violence and Suicide Risk and Self-Harm

To address the research question, multivariate logistic regression models (Table 2) examined the association between threat to post sexually explicit media and suicide risk and self-harm. In the model, a threat to post sexually explicit media without consent was associated with higher odds of reporting suicidal ideation (odds ratio [OR] = 1.84, confidence interval, CI [1.16, 2.92]), suicide plan (OR = 2.11, CI [1.31, 3.43]), suicide attempt (OR = 3.51, CI [2.02, 6.08]), and self-harm (OR = 1.96, CI [1.23, 3.12]). Being older was associated with lower odds of reporting suicide risk and self-harm and being assigned female sex at birth was associated with higher odds of reporting suicidal ideation, suicide planning, and self-harm. Models also controlled for sexual identity and race and ethnicity.

**Table 2.** Digital Sexual Violence (Threat to Post) and Suicide Risk.

	Suicidal Ideation		Suicidal Plan		Suicidal Attempt		Self-Harm	
	OR (CI)	*p*	OR (CI)	*p*	OR (CI)	*P*	OR (CI)	*p*
Threat	**1.84 (1.16–2.92)**	.010	**2.11 (1.31–3.43)**	.002	**3.51 (2.02–6.08)**	<.001	**1.96 (1.23–3.12)**	.005
Age	**0.84 (0.74–0.96)**	.007	**0.80 (0.69–0.93)**	.003	**0.78 (0.64–0.95)**	.013	**0.83 (0.74–0.94)**	.004
Female	**2.10 (1.50–2.92)**	<.001	**2.38 (1.58–3.60)**	<.001	1.37 (0.79–2.37)	.27	**2.32 (1.68–3.22)**	<.001
Sexual Identity								
Bisexual	1.09 (0.78–1.52)	.63	0.82 (0.55–1.22)	.33	1.12 (0.65–1.94)	.69	1.07 (0.77–1.49)	.68
Pansexual +	1.08 (0.68–1.71)	.77	1.31 (0.79–2.17)	.30	1.78 (0.91–3.45)	.09	1.53 (0.97–2.41)	.07
Another Identity	1.02 (0.64–1.65)	.90	0.88 (0.50–1.54)	.65	1.03 (0.46–2.28)	.94	1.25 (0.78–2.01)	.36
Race								
Latino/Hispanic	0.88 (0.58–1.34)	.55	0.98 (0.60–1.60)	.93	0.98 (0.50–1.92)	.95	0.90 (0.60–1.35)	.62
Black/African American	1.12 (0.68–1.85)	.66	1.49 (0.85–2.61)	.16	1.90 (0.96–3.75)	.07	0.64 (0.38–1.09)	.10
Asian/PI	0.79 (0.45–1.39)	.41	0.74 (0.36–1.51)	.41	0.35 (0.81–1.47)	.15	0.70 (0.40–1.23)	.22
Native American/AI/AN	1.67 (0.74–3.78)	.22	0.85 (0.30–2.38)	.76	0.71 (0.16–3.21)	.65	0.75 (0.33–1.72)	.50
Multi-racial	1.17 (0.75–1.82)	.49	1.15 (0.69–1.90)	.60	1.10 (0.54–2.20)	.41	1.14 (0.74–1.76)	.56

*Note*. Reference category for sex-at-birth is male, for sexual identity is gay/lesbian, for race is white/Caucasian. Threat = threat to post intimate or sexual content (photos, images, or videos) without one’s consent.

AI = American Indian; AN = Alaska Native; CI = confidence interval; OR = odds ratio; PI = Pacific Islander.

*p* = *p* value; bold indicates significance, *p* < .05.

Similarly, multivariate logistic regression models (Table 3) examined the association between nonconsensual posting of sexually explicit media and suicide risk and self-harm. Nonconsensual posting of sexually explicit media was associated with higher odds of reporting suicidal ideation (OR = 1.77, CI [1.04, 3.05]) and suicide attempt (OR = 2.15, CI [1.08, 4.28). Being older was associated with lower odds of reporting suicide risk and self-harm; being assigned sex assigned female at birth was associated with higher odds of reporting suicidal ideation, suicide planning, and self-harm, and identifying as Black or African American was associated with higher odds of reporting suicidal attempts.

**Table 3.** Digital Sexual Violence (Nonconsensual Posting) and Suicide Risk.

	Suicidal Ideation		Suicidal Plan		Suicidal Attempt		Self-Harm	
	OR (CI)	*p*	OR (CI)	*p*	OR (CI)	*p*	OR (CI)	*p*
Posted	**1.77 (1.04–3.05)**	.04	1.36 (0.74–2.49)	.32	**2.15 (1.08–4.28)**	.03	1.68 (0.98–2.88)	.06
Age	**0.85 (0.75–0.96)**	.008	**0.80 (0.70–0.93)**	.003	**0.79 (0.65–0.96)**	.03	**0.84 (0.74–0.95)**	.004
Female	**2.17 (1.56–3.03)**	<.01	**2.46 (1.63–3.70)**	<.001	1.54 (0.89–2.65)	.12	**2.36 (1.71–3.28)**	<.001
Sexual Identity								
Bisexual	1.07 (0.77–1.50)	.68	0.83 (0.56–1.23)	.36	1.09 (0.64–1.87)	.76	1.07 (0.77–1.48)	.71
Pansexual +	1.08 (0.68–1.71)	.74	1.35 (0.82–2.23)	.24	1.77 (0.92–3.41)	.09	1.56 (0.99–2.46)	.06
Another Identity	1.02 (0.64–1.65)	.92	0.88 (0.50–1.54)	.65	0.99 (0.45–2.18)	.99	1.25 (0.78–2.00)	.36
Race								
Latino/Hispanic	0.90 (0.60–1.37)	.63	0.98 (0.60–1.61)	.95	1.00 (0.51–1.95)	.99	0.92 (0.61–1.38)	.68
Black/African American	1.15 (0.70–1.89)	.59	1.46 (0.84–2.55)	.18	**1.95 (1.01–3.77)**	.047	0.65 (0.38–1.09)	.10
Asian/PI	0.80 (0.45–1.41)	.44	0.71 (0.34–1.45)	.35	0.32 (0.76–1.36)	.12	0.70 (0.40–1.23)	.21
Native American/AI/AN	170 (0.75–3.83)	.20	0.88 (0.32–2.45)	.81	0.76 (0.17–3.36)	.71	0.76 (0.33–1.75)	.53
Multi-racial	1.17 (0.75–1.82)	.10	1.11 (0.67–1.84)	.15	1.04 (0.52–2.09)	.91	1.15 (0.74–1.79)	.53

*Note*. Reference category for sex-at-birth is male, for sexual identity is gay/lesbian, for race is white/Caucasian. Posted = posted intimate or sexual content (photos, images, or videos) without one’s consent.

AI = American Indian; AN = Alaska Native; CI = confidence interval; OR = odds ratio; PI = Pacific Islander.

*p* = *p* value; bold indicates significance, *p* < .05.

## Discussion

This study extends work that has explored the associations between digital sexual violence and suicide risk in youth generally (Medrano et al., 2018; Wachs et al., 2021) and among sexual minority youth, who reported elevated relative levels of digital sexual victimization (Patchin & Hinduja, 2020; Van Ouytsel et al., 2021) and suicidality (Fulginiti et al., 2020; Luk et al., 2021; Marshal et al., 2011). In our sample, a threat of someone posting their sexually explicit material was significantly associated with suicidal ideation, making a suicide plan, suicide attempt, and non-suicidal self-injury. Given that threats are made to persuade or coerce behavior, sexual minority adolescents who may not have disclosed their sexual identity to some people may be more susceptible to coercion to maintain their privacy. Stigma around sexting behaviors or sexual identity may also limit sexual minority adolescents’ access to social support if they do not feel able to discuss potential victimization with others. This feeling of limited or no options may contribute to the increased rates of suicidality and self-injury reported in our sample among sexual minority adolescents who also reported that others had made digital sexual violence threats against them.

Likewise, having explicit material about the participant posted was associated with increased suicidal ideation and suicide attempt, though we did not find an association with making a suicide plan and non-suicidal self-injury. Enacted victimization differs from threats in that participants are faced with responding to an event, rather than the possibility of an event. It may be that the consequences of enacted victimization are worse, or not as bad, as they would have imagined. Similarly, it may be easier for bystanders to intervene on behalf of a victim of digital sexual violence than for a potential victim. Regardless, that the experience of nonconsensual posting of one’s sexually explicit material is associated with suicidal ideation and suicide attempt is concerning. Consistent with other research on suicidality among sexual minority adolescents (Kann et al., 2018; Luk et al., 2021), age was negatively associated with suicidality, such that younger participants exhibited higher rates of all suicide items measured and participants assigned female sex at birth had higher rates of suicidal ideation, suicide planning, and self-injury, but not suicide attempt.

These findings underscore important considerations and future research directions. Given the associations of digital sexual violence, suicidality, and self-harm among sexual minority adolescents, more awareness of and interventions to prevent digital sexual violence may also work to reduce suicidality in this affected population. Messaging may need to be broad to reach those who may not report a violence experience or may conceal their sexual identity. Although suicidality often decreases with age, dating behaviors increase, so messaging and interventions should be tailored with developmental trajectories in mind. In addition, the trajectories of digital sexual violence among adolescents and comparative analyses by demographic subgroups may indicate additional tailoring for interventions and sensitize practitioners to important developmental and demographic considerations when working with adolescents at risk of suicidality, self-harm, and digital sexual violence.

### Limitations and Conclusion

This study had several limitations. The sample included only cisgender adolescents and did not include transgender or gender nonbinary adolescents, and further work is clearly needed to include this population that also experiences very high levels of suicidal behaviors (Srivastava et al., 2021). Internet survey research has distinct advantages, especially for reaching marginalized, geographically dispersed minority populations (Stern et al., 2020). We recruited a large sample of diverse sexual minority adolescents from both urban and rural areas of United States. However, Internet-based recruitment and data collection also have limitations and challenges. In terms of generalizability, our findings are limited to adolescents who have access to the internet and online spaces. Although there is now significant evidence that the demographic and behavioral characteristics of those recruited online are similar to those recruited through more traditional, in-person venues. The authors have recoded the responses on suicide attempts and self-harm from continuous to dichotomous, which may restrict the interpretation of these variables.

Despite these limitations, to our knowledge, this paper is the first to examine the relationship between digital sexual violence and suicide risk in a nationwide sample of cisgender sexual minority adolescents. Given pervasive homonegative social and political climates in many areas, sexual minority adolescents will continue to experience digital sexual violence and victimization in less supportive environments, resulting in negative behavioral health outcomes. Suicide prevention efforts with adolescents must be responsive to the needs of sexual minority adolescents and the changing landscape of sexual violence in digital space.
